# Src inhibitors act through different mechanisms in Non-Small Cell Lung Cancer models depending on EGFR and RAS mutational status

**DOI:** 10.18632/oncotarget.4636

**Published:** 2015-08-03

**Authors:** Luigi Formisano, Valentina D'Amato, Alberto Servetto, Simona Brillante, Lucia Raimondo, Concetta Di Mauro, Roberta Marciano, Roberta Clara Orsini, Sandro Cosconati, Antonio Randazzo, Sarah J. Parsons, Nunzia Montuori, Bianca Maria Veneziani, Sabino De Placido, Roberta Rosa, Roberto Bianco

**Affiliations:** ^1^ Department of Clinical Medicine and Surgery, University of Naples “Federico II”, Naples, Italy; ^2^ DiSTABiF, Second University of Naples, Caserta, Italy; ^3^ Department of Pharmacy, University of Naples “Federico II”, Naples, Italy; ^4^ Department of Microbiology, Immunology & Cancer Biology, Cancer Center, University of Virginia Health System, Charlottesville, Virginia, USA; ^5^ Department of Translational Medical Sciences, University of Naples Federico II, Naples, Italy; ^6^ Department of Molecular Medicine and Medical Biotechnologies, University of Naples “Federico II”, Naples, Italy

**Keywords:** Src, NSCLC, EGFR inhibitors, MEK inhibitors, drug resistance

## Abstract

Resistance to the EGFR tyrosine kinase inhibitors (TKIs) gefitinib and erlotinib, often related to Ras or secondary EGFR mutations, is a relevant clinical issue in Non-Small Cell Lung Cancer (NSCLC). Although Src TK has been involved in such resistance, clinical development of its inhibitors has been so far limited.

To better define the molecular targets of the Src TKIs saracatinib, dasatinib and bosutinib, we used a variety of *in vitro/in vivo* studies.

Kinase assays supported by docking analysis demonstrated that all the compounds directly inhibit EGFR TK variants. However, in live cells only saracatinib efficiently reduced EGFR activation, while dasatinib was the most effective agent in inhibiting Src TK. Consistently, a pronounced anti-proliferative effect was achieved with saracatinib, in EGFR mutant cells, or with dasatinib, in wt EGFR/Ras mutant cells, poorly dependent on EGFR and erlotinib-resistant. We then identified the most effective drug combinations to overcome resistance to EGFR inhibitors, both *in vitro* and in nude mice: in T790M EGFR erlotinib-resistant cells, saracatinib with the anti-EGFR mAb cetuximab; in Ras mutant erlotinib-resistant models, dasatinib with the MEK inhibitor selumetinib.

Src inhibitors may act with different mechanisms in NSCLCs, depending on EGFR/Ras mutational profile, and may be integrated with EGFR or MEK inhibitors for different cohorts of NSCLCs.

## INTRODUCTION

Non-small cell lung cancer (NSCLC) is the leading cause of cancer-related death worldwide [1]. The epidermal growth factor receptor (EGFR) is a well characterized mutated oncogene in NSCLC: mutations (exon 19 deletions, exon 18 variants and L858R substitution in exon 21) leading to constitutive kinase activation are found in ∼10–20% of cases in western countries and are associated predominantly with adenocarcinoma histology [2–4]. EGFR-mutated tumors depend on EGFR signaling for their proliferation and survival: consistently, the EGFR tyrosine kinase inhibitors (TKIs) gefitinib, erlotinib and afatinib represent a relevant therapeutic option for NSCLC patients with EGFR activating mutations [5–7]. Indeed, mutant EGFR kinase binds the TKIs more tightly than the wild-type (wt). Unfortunately, *de novo* resistance to TKIs is often observed and virtually all patients who initially respond ultimately develop acquired resistance. Mechanisms of *de novo* resistance include K-RAS (15–25%) or B-RAF (2–3%) mutations, alterations in the exon 20 of the EGFR (∼5%), such as the T790M substitution, activation of phosphoinositide-3-Kinase (PI3K)/Akt or insulin-like growth factor 1 receptor (IGF-1R) signaling. Acquired resistance may depend on second-site EGFR mutations (50%; i.e. T790M), MET or HER2 amplification, PI3K mutations, activation of AXL, PI3K or IGF-1R pathways, small cell transformation [8–11]. Alternative strategies for the treatment of patients after failure of EGFR TKIs are considered high-priority areas of research [12].

Although EGFR is generally activated through ligand binding and autophosphorylation of its cytoplasmic tail, it is well established that Src non-receptor TK, one of the EGFR downstream transducers, can transactivate EGFR by phosphorylating tyrosine 845 (Y845); this event may contribute to full receptor activation [13]. Based on this evidence, TKIs acting on Src family kinases (SFKs) such as saracatinib and dasatinib have been proposed as therapeutic agents for NSCLC; these small molecules ATP competitors can inhibit kinases beyond the SFKs, and off-target effects could have biological relevance. Some studies have described the effect of saracatinib [14, 15] or dasatinib [16] on cancer cell lines carrying EGFR TK variants, either wt or mutants. However, disappointing results from phase I/II clinical trials have so far delayed the clinical development of these drugs. Although all studies, conducted in prospectively unselected patients with advanced NSCLC, showed poor activity of Src inhibitors either in first and subsequent lines of therapy, some isolated clinical response have been reported [17–20].

In the present study, we attempted to clarify the possible role of Src inhibitors in the context of NSCLC therapy. To this purpose, we used a variety of *in vitro*/*in vivo* assays aimed at better defining the molecular targets of saracatinib, dasatinib and bosutinib, the three most clinically investigated Src TKIs. Moreover, we tried to suggest the optimal combination regimens and the clinical settings where the different anti-Src agents may better exert their antitumor activity.

## RESULTS

### Src inhibitors saracatinib, dasatinib and bosutinib inhibit EGFR tyrosine kinase activation

Given the conflicting reports concerning the capability of anti-Src agents to directly inhibit EGFR tyrosine kinase (TK) activity [14–16, 21, 22], we performed an *in vitro* kinase assay comparing the effect of the Src inhibitors saracatinib, dasatinib and bosutinib with that of erlotinib on different EGFR TK variants, both wt and mutant. As indicated in Table 1, all the compounds inhibited EGFR variants, but with varying IC_50_ values compared to erlotinib. Overall, dasatinib was slightly less effective than the other agents in this assay.

**Table 1:** Effect of Src inhibitors on EGFR TK catalytic activity

	EGFR d746-750	EGFR d747-749 A750P	EGFR d747-752 P753S	EGFR d752-759	EGFR G719C	EGFR G719S	EGFR L858R	EGFR L861Q	EGFR T790M	EGFR T790M/L858R	EGFR wt
Compound	IC_50_ (nM)	IC_50_ (nM)	IC_50_ (nM)	IC_50_ (nM)	IC_50_ (nM)	IC_50_ (nM)	IC_50_ (nM)	IC_50_ (nM)	IC_50_ (nM)	IC_50_ (nM)	IC_50_ (nM)
Saracatinib	13.1	13.3	10.0	36.2	9.77	13.5	9.40	12.3	769	802	11.0
Dasatinib	54.7	64.0	61.2	138	24.8	21.7	28.1	25.5	3100	2790	25.4
Bosutinib	2.53	5.80	3.47	23.7	2.59	2.25	2.29	3.95	145	214	3.17
Erlotinib	1.56	2.40	1.57	2.33	2.30	2.22	1.62	1.24	200	999	1.01

**Figure 1:**
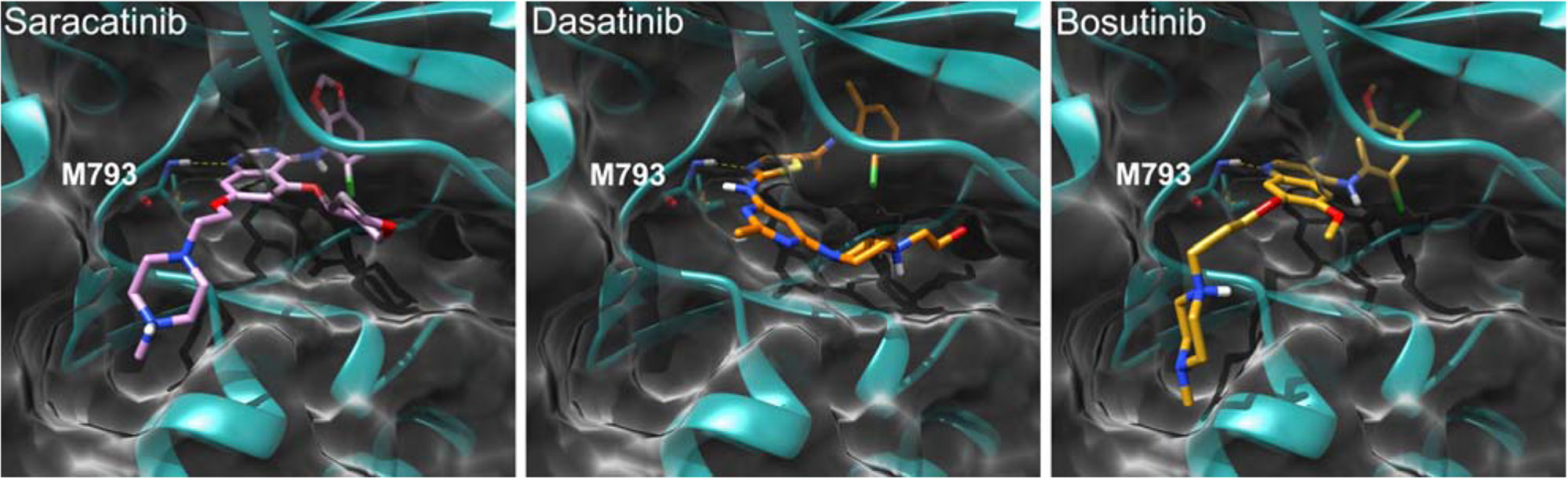
Binding mode of saracatinib, dasatinib, and bosutinib in the EGFR kinase domain (PDB 2ITT) as predicted by docking calculations The protein is depicted as cyan and white ribbons and surface, respectively. Key hinge region residue M793 is depicted as cyan sticks. Saracatinib, dasatinib, and bosutinib are represented as pink, orange, and yellow sticks, respectively. H-bond interactions are represented as dashed yellow lines.

Compound concentrations in the assay from 0.3 nM to 10 μM, semi-log steps; singlicate measurement Ranking of IC50 values: 
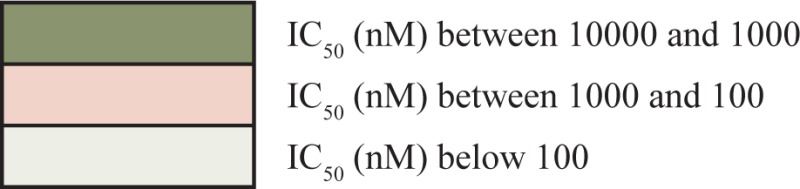

To better clarify how Src inhibitors could exert a direct effect on EGFR, we evaluated whether these compounds could adapt in the EGFR kinase domain binding site. As shown in Figure 1, docking results demonstrated that all the three compounds are able to settle in the enzyme ATP active site, establishing several interactions with the protein. A well-oriented hydrogen-bond with the M793 residue of the hinge region is formed with all the compounds, further substantiating their ability to act as typical EGFR TKIs.

### Src inhibitors exert different effects on human NSCLC cell lines

To further examine the inhibitory effects of the drugs, we used a panel of NSCLC cell lines with different levels of EGFR-dependent signaling activation and different degree of sensitivity against EGFR inhibitors. As shown in Table 2, this panel includes two cell lines with EGFR activating mutations (A746_A750del), PC-9 and HCC827 [23]; cell lines with wt EGFR, some of which harbor Ras and/or PI3K mutations and are thus resistant to anti-EGFR drugs (Calu3, H1299, H460, A549, GLC-82); and H1975 cells, containing a double EGFR mutation (L858R/T790M) that confers resistance to EGFR inhibitors [24]. We also included Calu3-ER, a cell line with acquired resistance to erlotinib, obtained from Calu3 cells through a validated protocol of *in vivo*/*in vitro* selection [25]; Calu3-ER cells display a significant increase in the expression of activated, phosphorylated MAPK compared to Calu-3 [26]. The above described NSCLC cells showed different levels of activation of EGFR-dependent signaling molecules such as Src, Akt and MAPK (Supplementary Figure S1). The observed levels are consistent with the mutational status of the cell lines and with previous data [26, 27].

**Table 2:** Mutational status and sensitivity to EGFR inhibitors of NSCLC cell lines

Cell line	EGFR	K-Ras	N-Ras	PI3K	Erlotinib (concentration producing 50% survival inhibition, μM)	Cetuximab (concentration producing 50% survival inhibition, μM)
PC9	A746_A750del *				0.01–0.1	1.4–3.5
HCC827	A746_A750del *				0.1	0,07
Calu3					2,5–5	3.5
Calu3-ER					>5	>3.5
H1299			Q61K		5	1.4
H460		Q61H		E545K	>5	>3,5
A549		G12S		Q546K	>5	>3.5
GLC-82				H1047R	>5	3.5
H1975	L858R/T790M				>5	3.5

* Exon 19 deletion

**Figure 2:**
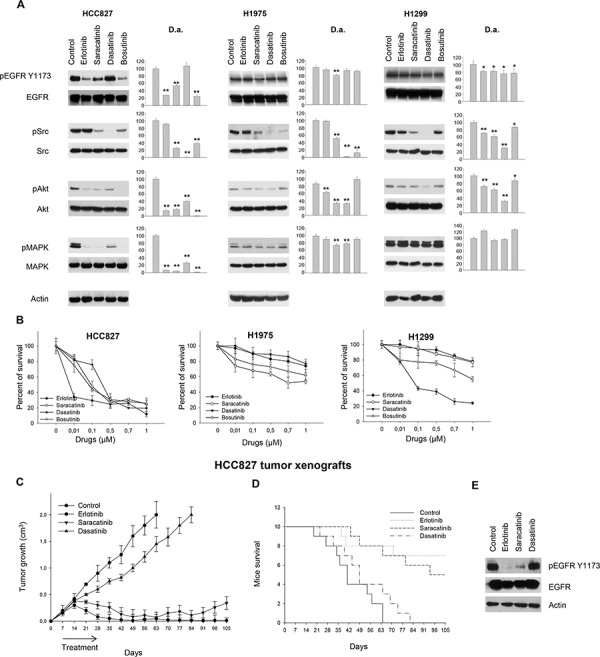
Effects of Src inhibitors on signal transduction and survival of human NSCLC cell lines sensitive or resistant to erlotinib **A.** Western blot analysis of protein extracts from HCC827, H1975 and H1299 cells treated for 3 hours with erlotinib, saracatinib, dasatinib or bosutinib (1 μM). Densitometric analysis (D.a.): The relative optical density of phospho-protein levels normalized to total protein levels is shown as histograms. *, 2-sided *P* < 0.05 *versus* control; **, 2-sided *P* < 0.01 *versus* control. **B.** Percent of survival of HCC827, H1975 and H1299 cells treated for 72 hours with erlotinib, saracatinib, dasatinib or bosutinib (1 μM), as measured by MTT assay. Data represent the mean (± SD) of three independent experiments, each performed in triplicate. Bars, SDs. **C.** After 7 days following subcutaneous injection of HCC827 cells, mice were randomized (10/group) to receive erlotinib, saracatinib or dasatinib, as described in the Methods section. The one-way ANOVA test was used to compare tumor sizes among treatment groups at the median survival time of the control group (42 days). Results are statistically significant for all the drugs vs control (*P* < 0.0001). Bars, SDs. **D.** Median survival was not statistically significant for dasatinib versus control (log-rank test); mouse groups treated with erlotinib or saracatinib did not reach a median survival, since 70% and 50% of animals were still alive at the end of the experiment, respectively. **E.** Western blot analysis was performed on total lysates from tumor specimens of two mice sacrificed on day 14. Tumors derived from each treatment group were pooled during lysis to obtain a single specimen.

When we tested Src inhibitors on these NSCLC models in comparison with erlotinib, we found that the three compounds have different effects on EGFR-dependent signal transduction. As shown in Figure 2A, saracatinib was the most efficient, among the tested compounds, in reducing EGFR phosphorylation on Y1173, a well-known autophosphorylation site; this effect was evident in EGFR mutant HCC827 cells. Surprisingly, the effect was detected also in the EGFR double mutant H1975 cells, although at a very slight degree. The reduction of pEGFR Y1173 was not found in cells harboring wt EGFR. Similar results were obtained in the other NSCLC cells tested (Supplementary Figure S2A); H460 cells were poorly sensitive to all the tested drugs because of low EGFR and Src activation (data not shown). In all cell lines, dasatinib was the most effective agent at inhibiting Src TK activity (Figure 2A and Supplementary Figure S2A). In cells expressing wt EGFR (H1299, Calu3, Calu3-ER, and A549), dasatinib was more effective at inhibiting Akt, while in cells with mutant EGFR, a greater inhibition of this transducer was observed with saracatinib and/or bosutinib (Figure 2A and Supplementary Figure S2A).

Consistent with data from Western blot analysis, in erlotinib-sensitive cells with EGFR-activating mutations (PC-9 and HCC827) saracatinib showed anti-proliferative effects that correlated with simultaneous EGFR/Src inhibition. Also in the EGFR double mutant model (H1975) saracatinib was slightly more effective than the other two compounds at inhibiting proliferation. Conversely, in wt EGFR/Ras mutant cells (H1299 and A549) that are poorly dependent on EGFR activation and thus erlotinib resistant, dasatinib was the most efficient inhibitor of cell proliferation (Figure 2B, Supplementary Table S1 and Supplementary Figure S2B). Consistent with this finding was our observation that the levels of Src activation in NSCLC cells are better associated with sensitivity to dasatinib than to saracatinib or bosutinib. In fact, linear regression analysis [28] demonstrated that high levels of Src activation in NSCLC cells significantly correlate with high sensitivity to dasatinib (*P* = 0.0382). This correlation was not found with saracatinib or bosutinib (*P* > 0.05) (Supplementary Figure S3).

Comparative growth inhibition was also studied in HCC827 tumor xenografts, as shown in Figure 2C. On day 63 (9 weeks after tumor cells injection) all tumors in control group reached the maximum allowed size of about 2 cm^3^. At this time point, dasatinib produced a tumor growth inhibition of about 27%, while erlotinib- and saracatinib-treated mice appeared to be tumor free. These latter agents maintained a potent antitumor activity until the end of the experiment, with about 99% and 83% of growth inhibition for erlotinib and saracatinib, respectively (Figure 2C). Consistently, as shown in Figure 2D, mice treated with erlotinib and saracatinib did not reach a median survival, since 70% and 50% of the mice were still alive at the end of the experiment, respectively. Treatments were well tolerated; no weight loss or other signs of acute or delayed toxicity were observed. Western blot analysis on tumor samples from mice sacrificed on day 14, after 1 week of treatment, demonstrated that saracatinib reduces EGFR phosphorylation similar to that observed with erlotinib, whereas dasatinib failed to do so (Figure 2E).

### Saracatinib exerts a direct, Src-independent effect on EGFR

We then investigated whether the effect of saracatinib on EGFR phosphorylation could be mediated by Src inhibition.

**Figure 3:**
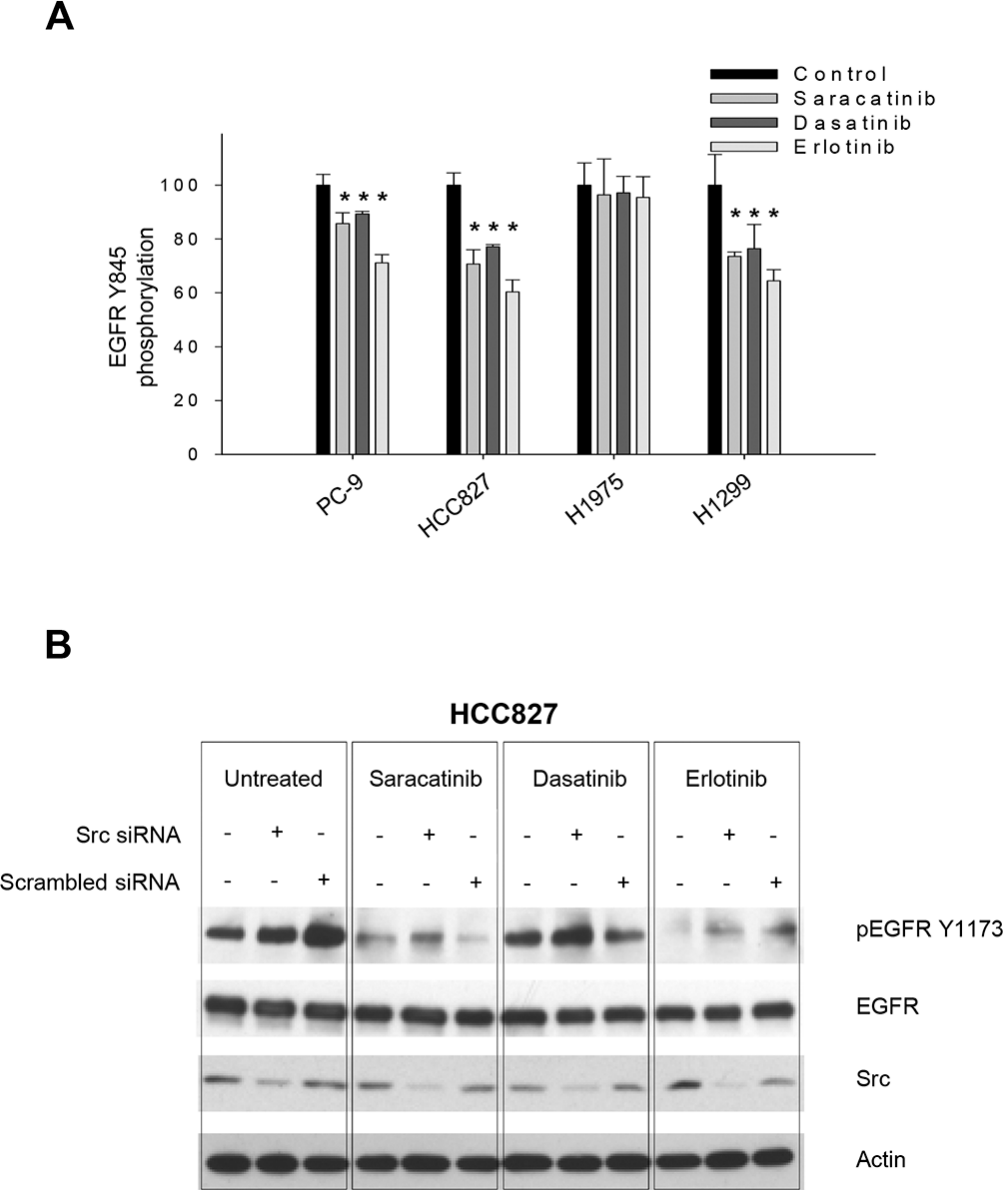
Correlation between drugs effect on human NSCLC cell lines and Src inhibition **A.** Percent of EGFR Tyr845 phosphorylation in PC-9, HCC827, H1975 and H1299 cells untreated (control) or treated for 3 hours with saracatinib, dasatinib or erlotinib (1 μM), as measured by the PathScan^®^ Phospho-EGF Receptor (Tyr845) Sandwich ELISA Kit. *, 2-sided *P* < 0.05 *versus* control. Data represent the mean (± SD) of three independent experiments, each performed in triplicate. Bars, SDs. **B.** Western blot analysis of protein expression in HCC827 cells transfected with a Src specific siRNA or with a negative, scrambled control, and then treated (24 hours after transfection) with saracatinib, dasatinib or erlotinib (1 μM) for 3 hours.

Since it is well known that Src can phosphorylate EGFR on Y845, we first verified the capability of Src inhibitors to interfere with this event through an ELISA assay measuring pEGFR Y845 levels. Saracatinib and dasatinib moderately inhibited EGFR phosphorylation on Y845 in PC-9, HCC827 and H1299 cells, while no effect was detected in H1975 cells (Figure 3A). Erlotinib also reduced pEGFR Y845 levels, as previously demonstrated [29, 30]. However, it has been reported that Src-mediated phosphorylation on EGFR Y845 does not affect EGFR autokinase activity [13], suggesting that EGFR Y1173 phosphorylation should not be decreased by Src inhibition, unless the inhibitors have cross-reactivity with the EGFR. Consistently, when Src expression was silenced in HCC827 cells with a specific siRNA, phosphorylation of EGFR on Y1173 was not reduced (Figure 3B). Saracatinib in the presence of Src siRNA maintained its capability to reduce EGFR Y1173 phosphorylation, thus supporting the hypothesis that the effect on EGFR was direct and not Src-mediated; the same result was obtained with erlotinib. Conversely, dasatinib had no effect on EGFR Y1173 phosphorylation also in the presence of Src silencing (Figure 3B). Levels of pEGFR Y845 was slightly reduced by Src siRNA. Saracatinib, dasatinib and erlotinib decreased EGFR phosphorylation at this site, as expected (Supplementary Figure S4).

### Cetuximab plus saracatinib is an effective combination in T790M EGFR erlotinib-resistant cells

**Figure 4:**
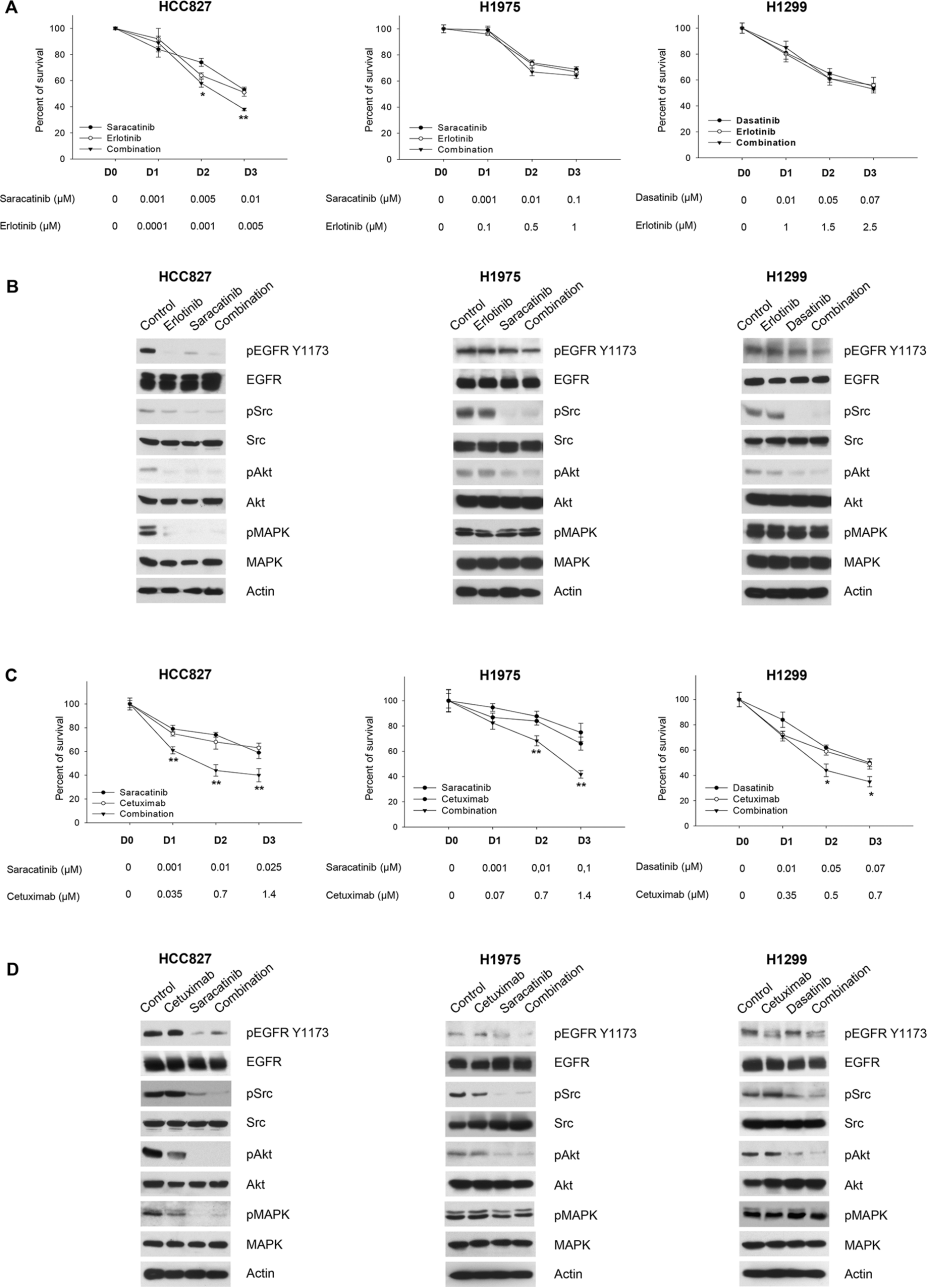
Effects of the combinations of EGFR and Src inhibitors on signal transduction and survival of human NSCLC cell lines **A.** Percent of survival of HCC827, H1975 and H1299 cells treated for 72 hours with different concentrations of saracatinib (for HCC827, H1975) or dasatinib (for H1299), alone or in combination with erlotinib, as measured by MTT assay. The doses of the two drugs used in combination have been chosen as equipotent at inhibiting cell survival. *, 2-sided *P* < 0.05 *versus* erlotinib alone; **, 2-sided *P* < 0.01 *versus* erlotinib alone. **B.** Western blot analysis of HCC827, H1975 and H1299 cells treated for 3 hours with saracatinib (for HCC827, H1975) or dasatinib (for H1299), alone or in combination with erlotinib. The maximum doses used in MTT assays have been chosen for Western blot analysis. **C.** Percent of survival of HCC827, H1975 and H1299 cells treated for 72 hours with different concentrations of saracatinib (for HCC827, H1975) or dasatinib (for H1299), alone or in combination with cetuximab, as measured by MTT assay. *, 2-sided *P* < 0.05 *versus* cetuximab alone; **, 2-sided *P* < 0.01 *versus* cetuximab alone. **D.** Western blot analysis of HCC827, H1975 and H1299 cells treated for 3 hours with saracatinib (for HCC827, H1975) or dasatinib (for H1299), alone or in combination with cetuximab. The maximum doses used in MTT assays have been chosen for Western blot analysis. Data represent the mean (± SD) of three independent experiments, each performed in triplicate. Bars, SDs.

To better define the role of tumor cell dependence on Src and EGFR, we tested Src inhibitors in combination with different classes of EGFR inhibitors at their equipotent doses. We first evaluated the combination of the most effective Src inhibitor (saracatinib for PC-9, HCC827 and H1975; dasatinib for H1299 cells) with the anti-EGFR drugs erlotinib (Figure 4A, 4B, Supplementary Table S2 and Supplementary Figure S5) or cetuximab (Figure 4C, 4D, Supplementary Table S2 and Supplementary Figure S5) by MTT assays and Western blot analysis. In EGFR-addicted cells (both erlotinib-sensitive PC-9 and HCC827 cells and, more importantly, erlotinib-resistant H1975 cells), the best among the two tested combinations was saracatinib plus cetuximab: it was able to efficiently reduce cell survival (Figure 4C, Supplementary Table S2 and Supplementary Figure S5C) and to interfere with EGFR and EGFR-dependent activation of signal transducers (Figure 4D and Supplementary Figure S5D). This result was supported by synergy quantification using the Chou-Talalay method [31]: all the combination index (CI) values are < 1 for saracatinib plus cetuximab, but not for saracatinib plus erlotinib (Supplementary Table S3). In order to show the superiority of saracatinib plus cetuximab in other models of T790M EGFR erlotinib-resistant cells, HEK 293 cells (physiologically not expressing EGFR receptor) were transfected with L858R/T790M mutant EGFR. The experiments confirmed the previous results (Supplementary Figure S6).

**Figure 5:**
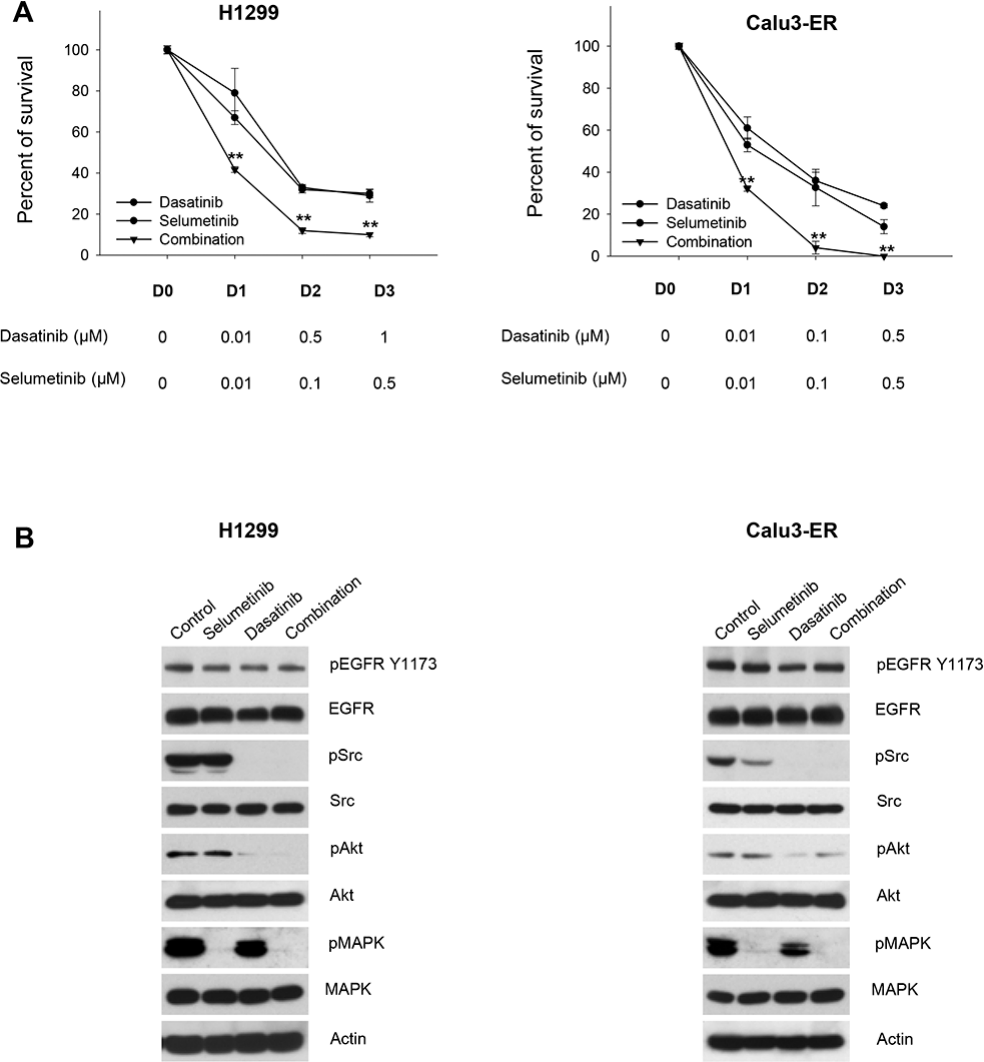
Effects of the combination dasatinib plus selumetinib on Ras-dependent human NSCLC models **A.** Percent of survival of H1299 and Calu3-ER cells treated for 72 hours with different concentrations of dasatinib, alone or in combination with selumetinib, as measured by MTT assay. **, 2-sided *P* < 0.01 *versus* selumetinib alone. **B.** Western blot analysis of H1299 and Calu3-ER cells treated for 3 hours with dasatinib, alone or in combination with selumetinib. The maximum doses used in MTT assays have been chosen for Western blot analysis.

In wt EGFR/Ras mutant cells (H1299) the combination of dasatinib plus cetuximab had a lower effect, either on cell survival and on signal transduction. Importantly, the combination did not reduce MAPK phosphorylation/activation (Figure 4C, 4D and Supplementary Table S2). Therefore, we hypothesized that MAPK signaling blockade could potentiate Src pharmacological inhibition in these cells. The combination of dasatanib with the MEK inhibitor selumetinib strongly inhibits cell survival in H1299 cells; similar effects were observed in Calu3-ER, a cell line with acquired resistance to erlotinib that shows a significant increase in the levels of activated, phosphorylated MAPK (Figure 5A and Supplementary Table S4). The combination was highly synergistic, as demonstrated by the very low combination index (CI) values calculated according to the Chou and Talalay method [31] using automated software (Supplementary Table S5). Consistently, dasatinib reduction of Src and Akt phosphorylation in conjunction with selumetinib suppression of MAPK phosphorylation/activation resulted in strong inhibition of signal transduction in both cell lines (Figure 5B). The effectiveness of selumetinib was not observed in the Ras mutant A549 cell line (data not shown), as previously reported [32].

### Cetuximab plus saracatinib is effective in mice xenografted with H1975 erlotinib-resistant tumors

**Figure 6:**
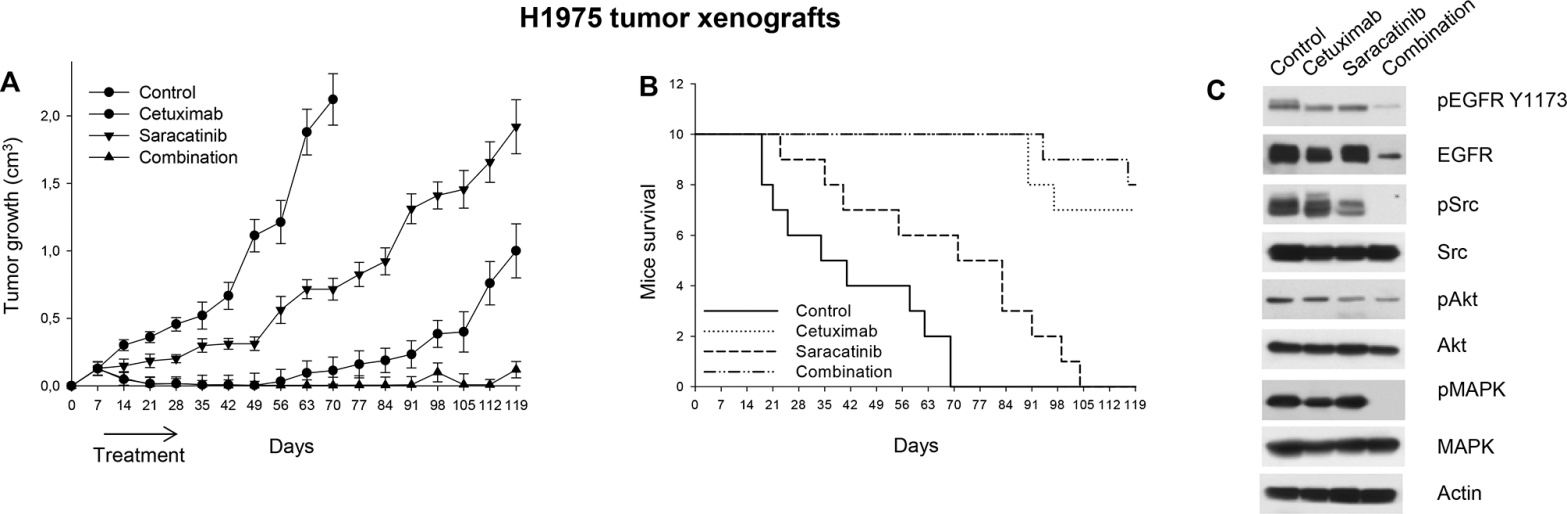
Effects of saracatinib plus cetuximab combination on tumor growth, survival and signal transduction of mice xenografted with H1975 erlotinib-resistant tumors **A.** After 7 days following subcutaneous injection of H1975 cells, mice were randomized (10/group) to receive cetuximab, saracatinib or their combination, as described in the Methods section. The one-way ANOVA test was used to compare tumor sizes among treatment groups at the median survival time of the control group (38.5 days). The results are statistically significant for the combination *versus* control or saracatinib (*P* < 0.0001). Bars, SDs. **B.** Median survival differences were statistically significant for the saracatinib versus control groups (log-rank test); mice treated with cetuximab or with the combination did not reach a median survival, since 70% and 80% of animals were still alive at the end of the experiment, respectively. **C.** Western blot analysis was performed on total lysates from tumor specimens of two mice sacrificed on day 14. Tumors derived from each treatment group were pooled during lysis to obtain a single specimen.

To further examine the inhibitory effects of cetuximab plus saracatinib we evaluated this combination *in vivo*, in erlotinib-resistant, EGFR double mutant H1975 tumor xenografts (Figure 6). On day 70 (10 weeks after tumor cells injection) all tumors of mice in the control group reached the maximum allowed tumor size of about 2 cm^3^. At this time point, saracatinib produced a tumor growth inhibition of about 66%, while cetuximab and the combination reduced tumor growth about 95%. Tumors of saracatinib-treated mice reached the size of 2 cm^3^ on day 119, while those of mice treated with cetuximab or cetuximab plus saracatinib never reached this size. The combination of cetuximab and saracatinib caused a long-lasting cooperative antitumor activity, as evidenced by 95% growth inhibition (*vs* 53% of cetuximab alone) on day 119 (Figure 6A). Consistently, as shown in Figure 6B, mice treated with the combination did not reach a median survival, since 80% of the mice were still alive at the end of the experiment. Treatments were well tolerated; no weight loss or other signs of acute or delayed toxicity were observed. Western blot analysis of tumor samples from mice sacrificed on day 14, after 1 week of treatment, demonstrated that cetuximab plus saracatinib efficiently interferes with EGFR-dependent signal transduction by reducing phosphorylation/activation of EGFR, Src, Akt and MAPK. The effect on EGFR is probably potentiated by EGFR down-regulation due to cetuximab-induced internalization (Figure 6C), as previously reported [24].

## DISCUSSION

Src inhibitors have been suggested as promising agents for NSCLC, but disappointing results from clinical trials have so far delayed their clinical development [17–20]. Major limitations of these studies may have been the enrolment of molecularly unselected patients with advanced NSCLC, and the lack of predictive biomarkers of response. Populations of patients with different molecular features could benefit from the treatment with different Src inhibitors despite seemingly negative outcomes of these trials, as some clinical responses have been reported. In the present study, we attempted to better define the mechanisms of action of three Src TKIs (saracatinib, dasatinib and bosutinib) in order to aid evaluation of how these agents could be integrated into current NSCLC therapy.

To this purpose, we used a variety of *in vitro*/*in vivo* assays to clarify the molecular targets of the above cited agents. In fact, several studies have suggested that these ATP competitors can inhibit kinases beyond the SFKs, including EGFR TK variants, either wt or mutant [14–16]; however, this issue still remains controversial. By using an *in vitro* kinase assay we demonstrated that the three Src inhibitors used in this study are able to inhibit EGFR TK activity, albeit with different degrees of efficacy (dasatinib being the least effective EGFR TKI). When tested on human NSCLC cell lines, Src inhibitors showed different effects. In erlotinib-sensitive cells containing EGFR-activating mutants (PC-9 and HCC827), saracatinib showed the greatest anti-proliferative effects among the three inhibitors (Figure 2B). This agent was also efficient at inhibiting EGFR phosphorylation on Y1173 (a well-known autophosphorylation site) in these cells. Similar results were obtained *in vivo*, in HCC827 tumor xenografts, where saracatinib showed a potent and long-lasting antitumor activity as compared to dasatinib (Figure 2C–2E). These findings are of clinical interest, as a recent publication of a phase II clinical trial reported two partial responses out of 31 NSCLC patients treated with saracatinib monotherapy [20]. One of the responses was long lasting, and the tumor harbored an EGFR exon 19 deletion. More importantly, in our study, saracatinib was also shown to be an effective growth inhibitor of the EGFR T790M mutant model *in vitro* (H1975 cells, Figure 2B), exhibiting greater inhibition than erlotinib, bosutinib, or dasatinib. Conversely, in wt EGFR-containing cells (H1299, Calu3, and Calu3-ER, Figure 2B), dasatinib was the most effective agent at inhibiting cell proliferation, accompanied by efficient inhibition of Src and Akt. In fact, dasatinib was the most effective agent at inhibiting Src TK in all cell lines.

Saracatinib has been shown to inhibit purified EGFR variants in a kinase assay; therefore, we attempted to determine whether its effect on EGFR Y1173 phosphorylation observed in NSCLC cell lines may be mediated by Src inhibition. It is well known that Src can phosphorylate EGFR on Y845, but it has been reported that Src phosphorylation on this residue does not affect EGFR autokinase activity [13], suggesting that EGFR Y1173 phosphorylation should not be decreased by Src inhibition, unless the anti-Src agents have cross-reactivity with EGFR. Consistently, when we silenced Src expression in HCC827 cells, we found that phosphorylation of EGFR on Y1173 was not reduced. Treatment with saracatinib in presence of Src silencing maintained its capability to reduce EGFR Y1173 phosphorylation. These data suggest that the effect observed in NSCLC cell lines after saracatinib treatment is not strictly Src-mediated.

Altogether, these data suggest that the three compounds may act with different mechanisms in NSCLC cell lines: while dasatinib, as expected, functions exclusively via Src inhibition, saracatinib showed an additional mechanism of action based on a direct EGFR inhibition. Bosutinib seemed to have an intermediate behavior, with a certain degree of variability among the different cell lines. As further evidence for these distinctions in mechanism of action, levels of activated Src in NSCLC cells correlated with sensitivity to dasatinib more than to saracatinib or bosutinib.

In an attempt to overcome resistance to EGFR inhibitors used in the clinic, we explored several inhibitor combinations and settings where the different anti-Src agents may exert their antitumor activity. Based on our finding that saracatinib as a single agent had the greatest anti-proliferative activity among the three Src TKIs in EGFR mutant cells, we tested saracatinib in combination with erlotinib or cetuximab in *in vitro* MTT assays. We observed that, in EGFR-addicted cells (either erlotinib-sensitive or -resistant), saracatinib plus cetuximab was more effective than saracatinib plus erlotinib. While this result may have poor impact on erlotinib-sensitive NSCLCs, for which erlotinib monotherapy remains the best therapeutic strategy, it may have great relevance for erlotinib resistant NSCLCs. This result was confirmed in an additional model of T790M EGFR erlotinib-resistance, obtained by transfecting HEK 293 cells (physiologically not expressing EGFR receptor) with L858R/T790M mutant EGFR. Conversely, in wt EGFR/Ras mutant models, we found that a combination of dasatanib and the MEK inhibitor, selumetinib, efficiently reduced cell survival and signal transduction, producing a highly synergistic effect. These data are of relevance based on the promising efficacy of selumetinib plus docetaxel combination reported in a randomized phase 2 clinical trial for K-Ras mutant advanced NSCLC [33].

Xenograft studies with the erlotinib-resistant, EGFR double mutant H1975 cell line supported the *in vitro* result, with the saracatinib plus cetuximab combination showing a cooperative antitumor activity and prolonged mouse survival. Interestingly, the effect of saracatinib on EGFR was probably potentiated by EGFR down-regulation due to cetuximab-induced internalization, as previously reported [24]. Others have found that targeting EGFR by using two different classes of inhibitors, namely a monoclonal antibody directed against the extracellular portion of the receptor and a TKI able to interfere with ATP binding, is effective in *in vitro* models of NSCLC harboring the T790M mutation; together, these agents efficiently depleted both phosphorylated and total EGFR [24]. A combination of the irreversible EGFR TKI afatinib with cetuximab was tested in a phase Ib clinical trial in advanced EGFR-mutated NSCLC cancers, which had progressed after EGFR TKI therapy. Among 126 patients, a high objective response rate (overall 29%) was reported, with comparable numbers of T790M-positive and T790M-negative tumors responding [34]. This study supports the hypothesis that a significant proportion of tumors with acquired resistance to anti EGFR TKI remains dependent on EGFR signaling, and that combination therapies could have a significant impact in the clinical arena. However, it should be pointed out that afatinib and cetuximab possess overlapping toxicities (mainly diarrhea and skin rash) that could limit their use in patients. By contrast, Src inhibitors, at least based on data from completed clinical trials, seems to have a different toxicity profile [35]. It has been recently demonstrated that the antitumor activity of afatinib could be enhanced by the combination with dasatinib in NSCLC models with T790M-mediated resistance [36]. In addition, saracatinib offers the advantage of simultaneous EGFR/Src inhibition, possibly providing a further benefit in terms of tumor growth control and preventing the onset of Src-mediated resistance to EGFR inhibitors, as previously reported [37–39]. Therefore, our study may have relevant therapeutic implications for lung cancer patients, suggesting an effective strategy to overcome EGFR drug resistance.

In conclusion, we have demonstrated that different Src inhibitors act through different mechanisms in NSCLC models that are sensitive or resistant to erlotinib. This evidence may partially explain the failure of clinical trials with Src inhibitors in unselected NSCLC patients [17–20]. The off-target effect, particularly on EGFR mutant variants, could be a main strength of saracatinib in the setting of erlotinib-resistant patients with EGFR mutations. Moreover, based on their differential effects, Src inhibitors may better cooperate with EGFR or MEK inhibitors in NSCLCs. In this respect, since few therapeutic options are available for wt EGFR/Ras mutant NSCLC, the combination of dasatinib plus selumetinib may be a novel, potentially valuable strategy in the clinical setting.

## MATERIALS AND METHODS

### Compounds

Cetuximab was kindly provided by ImClone Systems. Erlotinib, saracatinib, dasatinib, bosutinib and selumetinib were purchased from Selleck Chemicals, Germany.

### *In vitro* EGFR kinase inhibition assay

The EGFR kinase inhibition assay on saracatinib, dasatinib and bosutinib was performed with an EGFR kinase mutant profile screening service by ProQinase (ProQinase GmbH, Freiburg, Germany). Briefly, compounds were tested at 10 different concentrations (standard range: 3 × 10^−10^M–1 × 10^−5^M; semilog dilutions) against human recombinant wt EGFR and ten EGFR mutants protein kinases, and IC_50_ values were calculated. IC_50_ values of EGFR reference inhibitor (gefitinib or erlotinib) were determined side-by-side. All assays were performed at the corresponding apparent ATP Km of each protein kinase using the radiometric ^33^PanQinase Assay™.

### Docking of the Src inhibitors saracatinib, dasatinib and bosutinib in the EGFR TK domain

The three-dimensional (3D) structures of saracatinib, dasatinib, and bosutinib were drawn using the Builder tool and generated with Ligprep module within the Schrodinger package (Schrödinger, LLC: New York, NY, 2009). The Glide program, of the same package, was used to dock these compounds into the X-ray crystal structure of the EGFR TK domain (PDB 2ITT) [40]. The receptor grid generation was performed for the box with a center in the putative binding site. The size of the box was determined automatically. The extra precision mode (XP) of Glide was used for the docking. The ligand scaling factor was set to 1.0. The geometry of the ligand binding site of the complex between the selected ligands and the enzyme was then optimized. The binding site was defined as the ligand and all amino acid residues located within 8 Å from the ligand. All the protein residues located within 2 Å from the binding site were used as a shell. The following parameters of energy minimization were used: OPLS2005 force field; water was used as an implicit solvent; a maximum of 5000 iterations of the Polak–Ribier conjugate gradient minimization method was used with a convergence threshold of 0.01 kJ 3 mol^−1^ Å^−1^. Molecular graphics were performed with the UCSF Chimera package. Chimera is developed by the Resource for Biocomputing, Visualization, and Informatics at the University of California, San Francisco (supported by NIGMS P41-GM103311) [41].

### Cell cultures

Human HCC827, Calu3, H460, H1299, A549 and NSCLC cell lines were obtained from the American Type Culture Collection (ATCC). PC-9 and GLC82 cell lines were kindly provided by Dr F. Morgillo. HEK 293 cell line was kindly provided by Dr N. Normanno. Calu3-ER (Erlotinib Resistant) cells were established as previously described [25]. All cell lines were authenticated using STR DNA profiling and maintained in RPMI medium supplemented with 10% heat-inactivated fetal bovine serum, 20 mM HEPES, pH 7.4, penicillin (100 IU/ml), streptomycin (100 μg/ml) and 4 mM glutamine (ICN, Irvine, UK) in a humified atmosphere of 95% air and 5% CO_2_ at 37°C.

### Cell survival assay (MTT)

Cells (10^4^ cells/well) were grown in 24-well plates and exposed for 72 hours to increasing doses of erlotinib, cetuximab, saracatinib, dasatinib or bosutinib, alone or in combination. The percentage of cell survival was determined using the 3-(4, 5-dimethylthiazol-2-yl)-2, 5-diphenyltetrazolium bromide (MTT). The drug concentration producing 50% survival inhibition was used as a marker of drug effect.

### Combination effect

The combination effect of two drugs used at their equipotent doses was evaluated based on the combination index (CI), calculated using Calcusyn software (Biosoft, Cambridge, UK) and defined as follows: CI = (D)_1_/(Dx)_1_ + (D)_2_/(Dx)_2_ + (D)_1_(D)_2_/(Dx)_1_(Dx)_2_, where: (Dx)_1_ is the dose of Drug 1 alone required to produce an X% effect; (D)_1_ is the dose of Drug 1 required to produce the same X% effect in combination with Drug 2; (Dx)_2_ is the dose of Drug 2 alone required to produce an X% effect; and (D)_2_ is the dose of Drug 2 required to produce the same X% effect in combination with Drug 1. The combination effect was defined as follows: CI < 1, synergistic effect; CI < 0.5, highly synergistic effect; CI = 1, additive effect; and CI > 1, antagonistic effect.

### Immunoprecipitation and western blot analysis

Total cell lysates from cell cultures or tumor specimens were resolved by 4–15% SDS-PAGE and probed with anti-human, polyclonal pEGFR Y1173, EGFR, monoclonal pMAPK, MAPK (Santa Cruz Biotechnology, Santa Cruz, CA), polyclonal pAkt, Akt, pSrc, Src (Cell Signaling Technologies, Beverly, MA) and monoclonal actin (Sigma-Aldrich, Milan). Immunoreactive proteins were visualized by enhanced chemiluminescence (Pierce, Rockford, IL, USA). Densitometric analyses were performed with Image J software (NIH).

### Analysis of EGFR phosphorylation on Tyr845

Levels on pEGFR Y845 in cell protein extracts was analyzed by using PathScan^®^ Phospho-EGF Receptor (Tyr845) Sandwich ELISA Kit (Cell Signaling Technologies, Beverly, MA), according to manufacturer's instructions.

### RNA interference

Small interfering RNA (siRNA) against Src was obtained from Ambion Life Technology (Grand Island, NY, USA). A nonsense sequence was used as negative control. For siRNA validation, cells were transfected with Src siRNAs (5 and 50 nmol/L) using Lipofectamine 2000 (Invitrogen) in Opti-MEM (Invitrogen); 24 or 48 hours after transfection, Western blot analysis for Src protein expression was done. For the assessment of siRNA effects on cell survival and signaling, cells were transfected with Src siRNA for 24 hours, then treated with saracatinib, dasatinib or erlotinib for 3 additional hours.

### Cells transfection with EGFR vectors

pcDNA 3.2 empty vector (e.v.) as well as pcDNA 3.2 vectors harbouring wt EGFR or the mutant variant L858R/T790M were kindly provided by Dr F. Morgillo. For validation of cell transfection, cells (HEK 293, physiologically not expressing EGFR) were transfected with empty vector (e.v.) *versus* pcDNA 3.2 vectors harbouring wt EGFR or the mutant variant L858R/T790M (5 μg) using Lipofectamine 2000 (Invitrogen) in Opti-MEM (Invitrogen); 24 or 48 hours after transfection, Western blot analysis for EGFR protein expression was done. For the assessment of the effects on cell survival and signaling, cells were transfected with L858R/T790M mutant EGFR for 24 hours, then treated with saracatinib, erlotinib or cetuximab for 48 hours (MTT assays) or 3 hours (Western blot analysis).

### Nude mice cancer xenograft models

Five week old Balb/c athymic (nu+/nu+) mice (Charles River Laboratories, Milan, Italy) maintained in accordance with institutional guidelines of the University of Naples Animal Care Committee were injected subcutaneously (s.c.) with HCC827 or H1975 cells (10^7^ cells/mice) re-suspended in 200 μL of Matrigel (CBP, Bedford, MA). After 7 days, tumors were detected and groups of 10 mice were randomized to receive: cetuximab 35 mg/kg intraperitoneally (i.p.) three times a week for 3 weeks, erlotinib 20 mg/kg via oral gavage three times a week for 3 weeks, saracatinib 50 mg/kg via oral gavage five times a week for 3 weeks, or dasatinib 20 mg/kg i.p. three times a week for 3 weeks. Animals treated with DMSO vehicle served as controls. Tumor volume (cm^3^) was measured using the formula π/6 × larger diameter × (smaller diameter)^2^ as previously reported [42].

### Statistical analysis

The results of *in vitro* experiments were analyzed by Student's *t* test and expressed as means and standard deviations (SDs) for at least three independent experiments performed in triplicates. The statistical significance was determined by one-way ANOVA and Dunnett's multiple comparison post-test regarding tumor growth, by log-rank test concerning mice survival [42]. Linear regression analysis for the correlation between drug concentration producing 50% survival inhibition and Src activation was performed by using Sigma Plot ver. 11.0, as reported [28]. All reported *P* values were two-sided. Analyses were performed with the BMDP New System statistical package version 1.0 for Microsoft Windows (BMDP Statistical Software, Los Angeles, CA).

## SUPPLEMENTARY FIGURES AND TABLES


