# Supplementary material for: Src inhibitors act through different mechanisms in Non-Small Cell Lung Cancer models depending on EGFR and RAS mutational status
Source: Oncotarget. 2015 Aug 3;6(28):26090–103. doi: 10.18632/oncotarget.4636 (PMC4694888; doi:10.18632/oncotarget.4636)
Supplement: Supplementary file 1 [file oncotarget-06-26090-s001.pdf]

SUPPLEMENTARY FIGURES AND TABLES

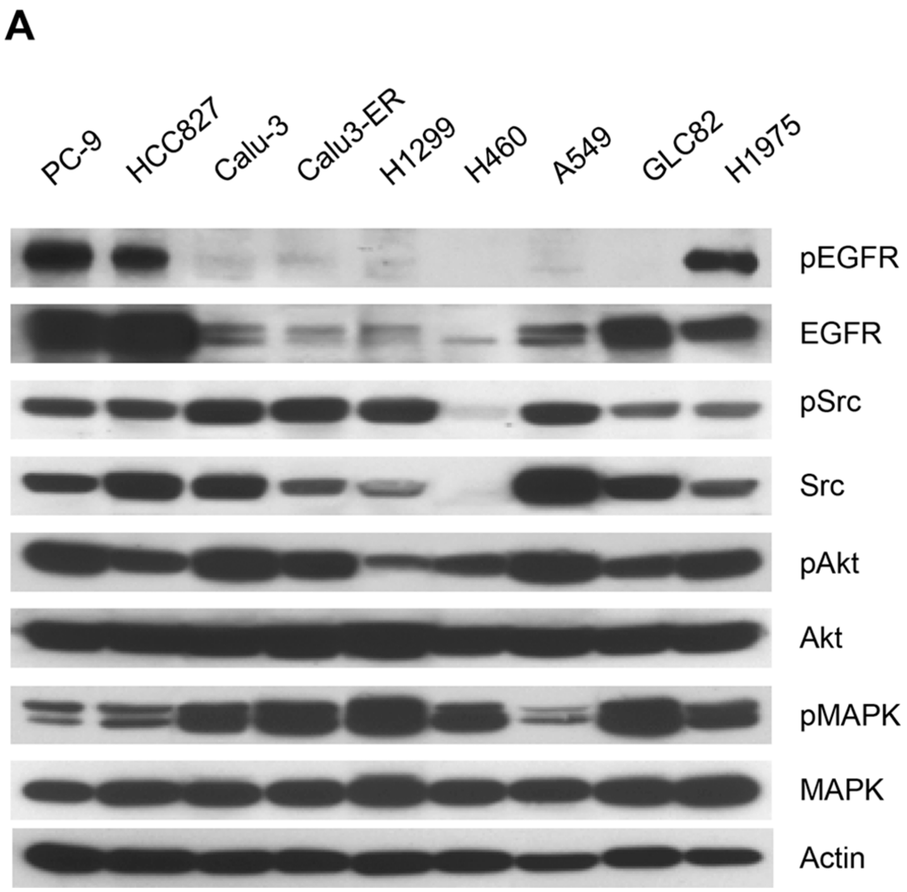

Supplementary Figure S1: EGFR-dependent pathway activation in a panel of human NSCLC cell lines. A. Western blot analysis of protein expression in a panel of human NSCLC cell lines.

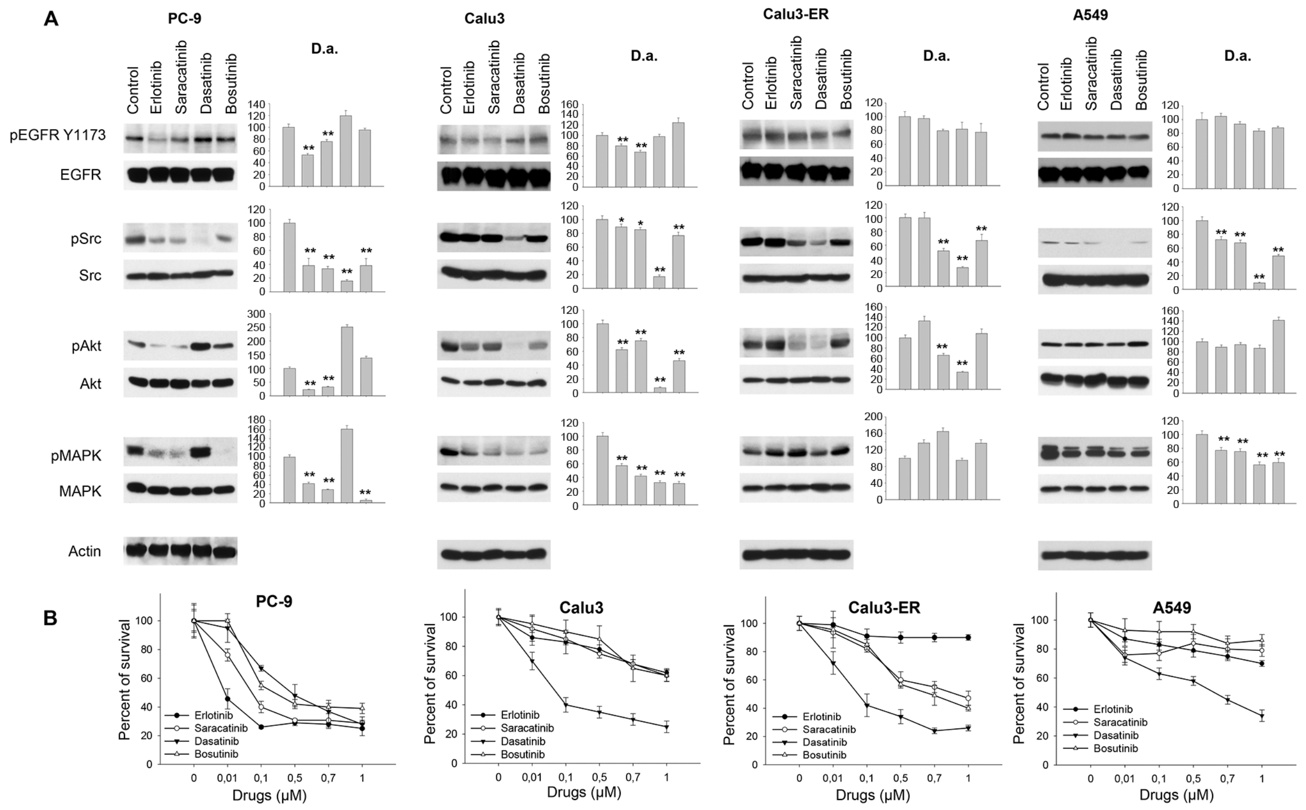

Supplementary Figure S2: Effects of Src inhibitors on signal transduction and survival of human NSCLC cell lines sensitive or resistant to erlotinib. A. Western blot analysis of protein expression in PC-9, Calu3, Calu3-ER and A549 cells treated for 3 hours with erlotinib, saracatinib, dasatinib or bosutinib (1 μM). Densitometric analysis (D.a.): The relative optical density of phospho-protein levels normalized to total protein level is shown as histograms. *, 2-sided $P < 0.05$ *versus* control; **, 2-sided $P < 0.01$ *versus* control. **B.** Percent of survival of PC-9, Calu3, Calu3-ER and A549 cells treated for 72 hours with erlotinib, saracatinib, dasatinib or bosutinib (1 μM), as measured by MTT assay. Data represent the mean (± SD) of three independent experiments, each performed in triplicate. Bars, SDs.

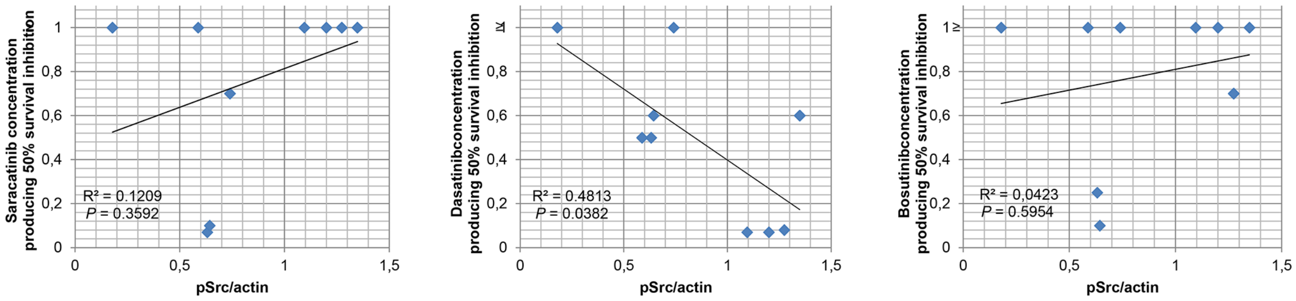

Supplementary Figure S3: Correlation between growth inhibition by saracatinib, dasatinib or bosutinib and Src baseline activation. The correlation between the concentration of saracatinib, dasatinib or bosutinib producing 50% growth inhibition and the ratio of p-Src/actin in 9 human NSCLC cell lines was shown. Protein expressions were measured by western blot (see Supplementary Figure S1) and analysed by ImageJ software. Linear regression analysis was performed by using Sigma Plot ver. 11.0.

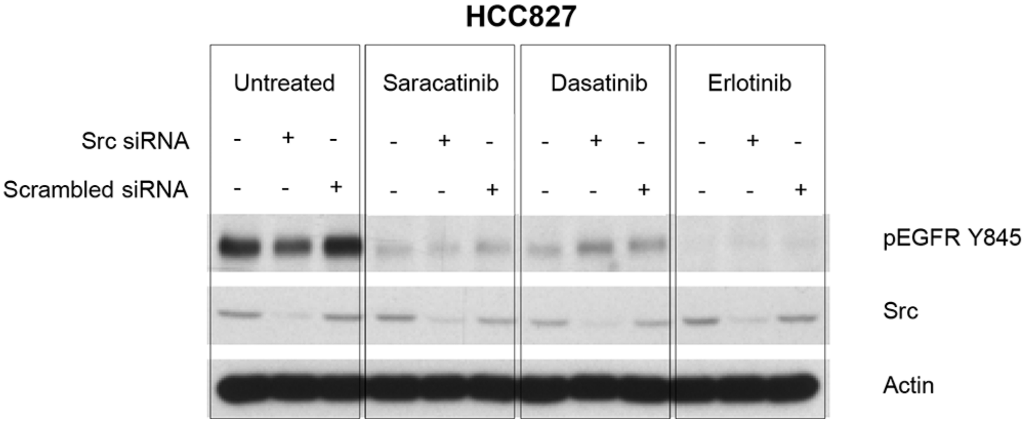

Supplementary Figure S4: Correlation between drugs effect on human NSCLC cell lines and Src inhibition. Western blot analysis of protein expression in HCC827 cells transfected with a Src specific siRNA or with a negative, scrambled control, and then treated (24 hours after transfection) with saracatinib, dasatinib or erlotinib (1 µM) for 3 hours.

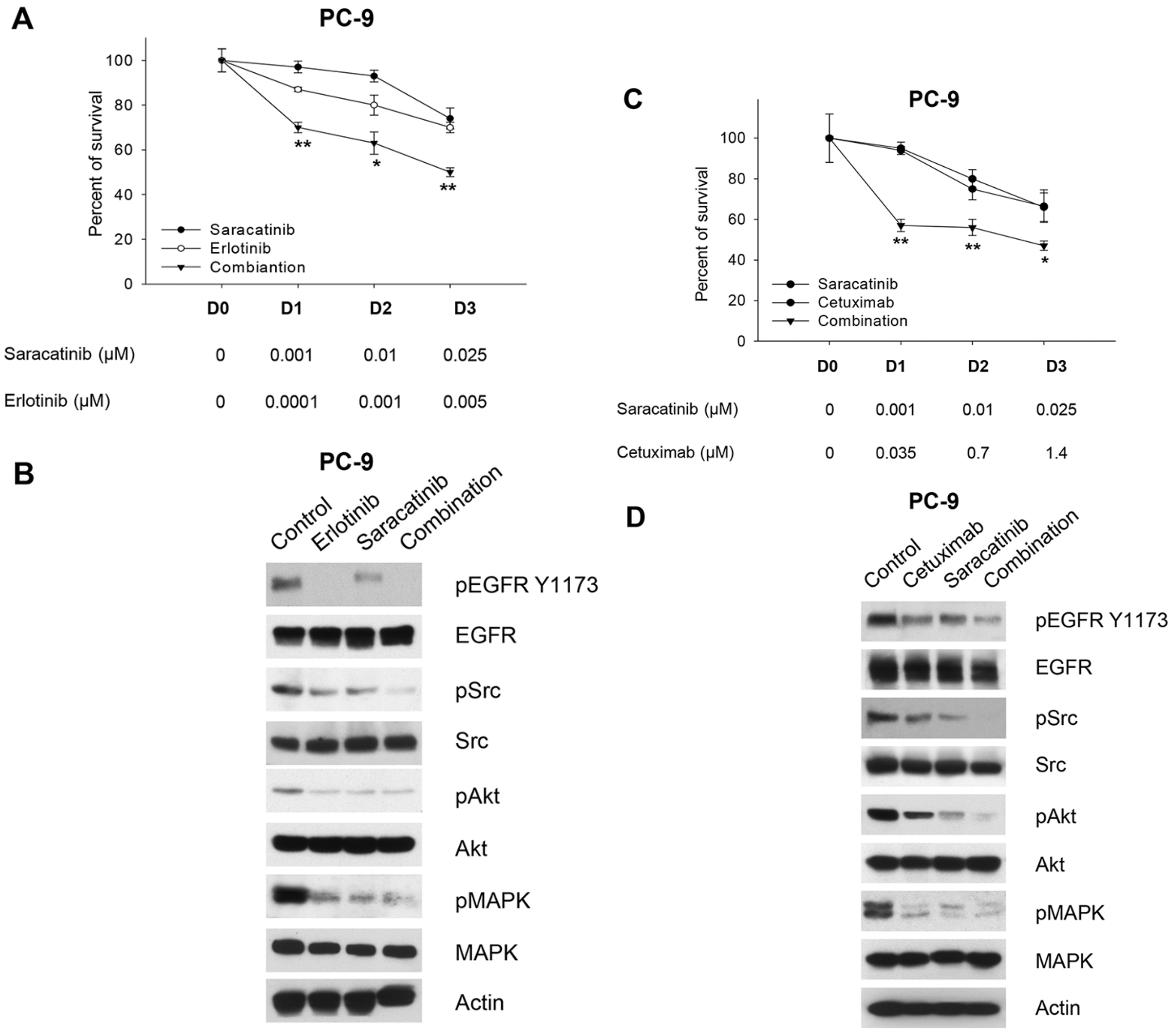

Supplementary Figure S5: Effects of the combinations of EGFR and Src inhibitors on signal transduction and survival of human NSCLC cell lines. A. Percent of survival of PC-9 cells treated for 72 hours with different concentrations of saracatinib, alone or in combination with erlotinib, as measured by MTT assay. The doses of the two drugs used in combination have been chosen as equipotent at inhibiting cell survival. *, 2-sided $P < 0.05$ *versus* erlotinib alone; **, 2-sided $P < 0.01$ *versus* erlotinib alone. **B.** Western blot analysis of PC-9 cells treated for 3 hours with saracatinib, alone or in combination with erlotinib. The maximum doses used in MTT assays have been chosen for Western blot analysis. **C.** Percent of survival of PC-9 treated for 72 hours with different concentrations of saracatinib, alone or in combination with cetuximab, as measured by MTT assay. *, 2-sided $P < 0.05$ *versus* cetuximab alone; **, 2-sided $P < 0.01$ *versus* cetuximab alone. **D.** Western blot analysis of PC-9 cells treated for 3 hours with saracatinib, alone or in combination with cetuximab. The maximum doses used in MTT assays have been chosen for Western blot analysis. Data represent the mean (± SD) of three independent experiments, each performed in triplicate. Bars, SDs.

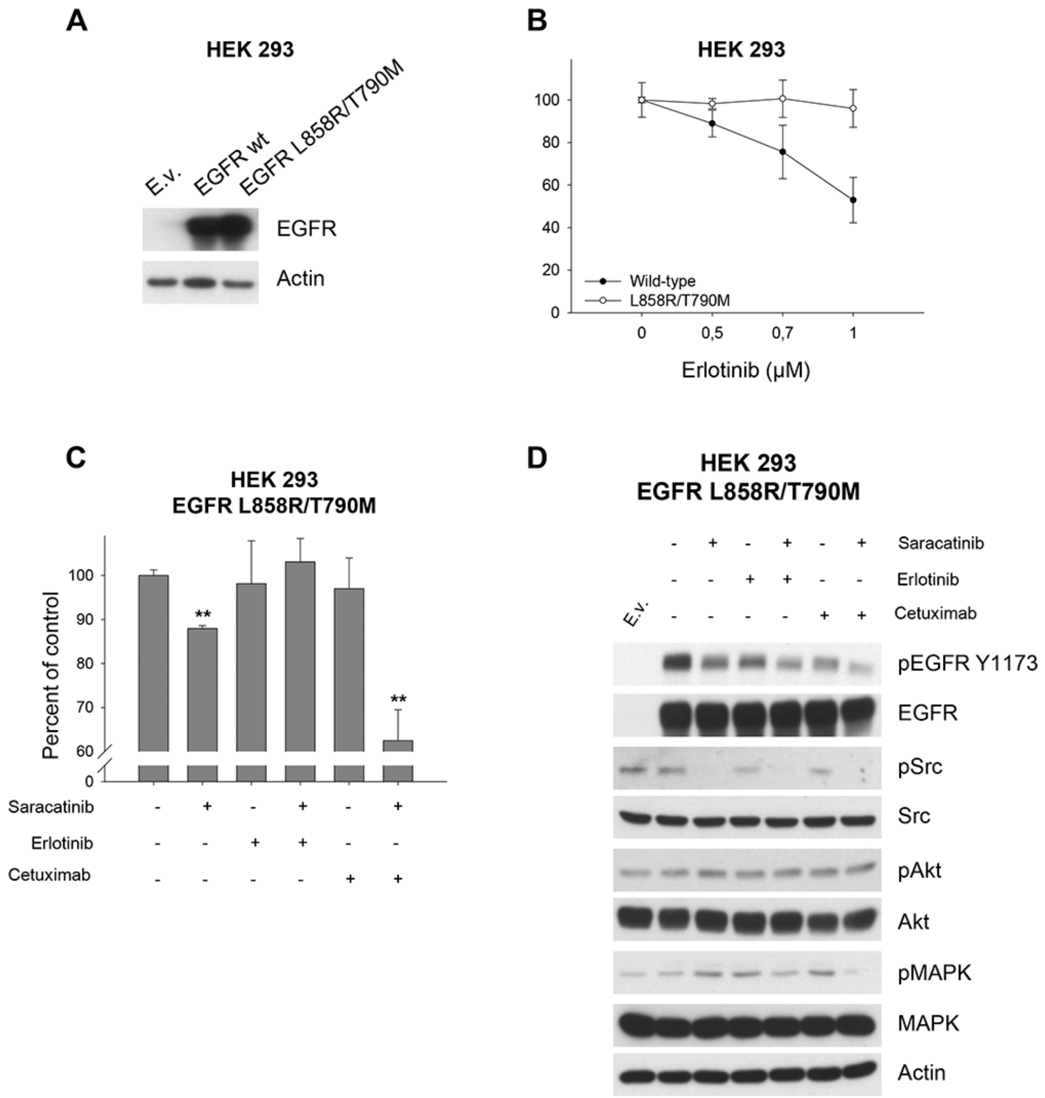

Supplementary Figure S6: Effects of the combinations of EGFR and Src inhibitors on signal transduction and survival of cells harbouring L858R/T790M mutant EGFR. A. Western blot analysis of HEK 293 cells 48 hours after transfection with pcDNA 3.2 empty vector (e.v.) *versus* pcDNA 3.2 vectors harbouring wt EGFR or the mutant variant L858R/T790M. **B.** Percent of survival of HEK 293 cells transfected with pcDNA 3.2 vector harbouring wt EGFR or the mutant variant L858R/T790M and treated for 48 hours with different concentrations of erlotinib, as measured by MTT assay. Differences between the slopes were statistically significant (wt versus L858R/T790M, $P < 0.01$). **C.** Percent of survival of HEK 293 cells transfected with pcDNA 3.2 vector harbouring L858R/T790M mutant EGFR and treated for 48 hours with saracatinib (1 µM), erlotinib (1 µM) or cetuximab (1.4 µM), alone and in combination, as measured by MTT assay. **, 2-sided $P < 0.01$ *versus* control. **D.** Western blot analysis of HEK 293 cells transfected with pcDNA 3.2 vector harboring L858R/T790M mutant EGFR and treated for 48 hours with saracatinib (1 µM), erlotinib (1 µM) or cetuximab (1.4 µM), alone and in combination. Data represent the mean (± SD) of three independent experiments, each performed in triplicate. Bars, SDs.

Supplementary Table S1: *P* values for survival inhibition by saracatinib, dasatinib and bosutinib (treatment vs control) in human NSCLC cell lines sensitive or resistant to erlotinib, as measured by MTT assay

HCC827

Drug	0.01 µM	0.1 µM	0.5 µM	0.7 µM	1 µM
Erlotinib	< 0.001	< 0.001	< 0.001	< 0.001	< 0.001
Saracatinib	0.023	< 0.001	< 0.001	< 0.001	< 0.001
Dasatinib	0.009	0.003	< 0.001	< 0.001	< 0.001
Bosutinib	0.032	< 0.001	< 0.001	< 0.001	< 0.001

H1975

Drug	0.01 µM	0.1 µM	0.5 µM	0.7 µM	1 µM
Erlotinib	0.463	0.140	0.012	0.004	0.012
Saracatinib	0.009	0.006	< 0.001	< 0.001	< 0.001
Dasatinib	1.000	0.041	0.068	0.051	0.002
Bosutinib	0.033	0.016	0.001	0.003	0.002

H1299

Drug	0.01 µM	0.1 µM	0.5 µM	0.7 µM	1 µM
Erlotinib	< 0.001	< 0.001	< 0.001	< 0.001	< 0.001
Saracatinib	0.032	0.001	< 0.001	< 0.001	< 0.001
Dasatinib	0.536	0.002	0.001	< 0.001	< 0.001
Bosutinib	1.000	< 0.001	< 0.001	< 0.001	< 0.001

PC-9

Drug	0.01 µM	0.1 µM	0.5 µM	0.7 µM	1 µM
Erlotinib	1.000	0.216	0.131	0.011	0.002
Saracatinib	0.640	0.354	0.018	0.009	0.007
Dasatinib	0.002	< 0.001	< 0.001	< 0.001	< 0.001
Bosutinib	0.009	0.016	0.002	0.005	< 0.001

Calu3

Drug	0.01 µM	0.1 µM	0.5 µM	0.7 µM	1 µM
Erlotinib	0.038	0.041	0.005	0.001	< 0.001
Saracatinib	0.233	0.015	0.002	< 0.001	< 0.001
Dasatinib	0.003	< 0.001	< 0.001	< 0.001	< 0.001
Bosutinib	0.375	0.14	0.065	0.004	< 0.001

Calu3-ER

Drug	0.01 µM	0.1 µM	0.5 µM	0.7 µM	1 µM
Erlotinib	0.819	0.092	0.054	0.041	0.032
Saracatinib	0.145	0.005	< 0.001	< 0.001	< 0.001
Dasatinib	0.007	< 0.001	< 0.001	< 0.001	< 0.001
Bosutinib	0.606	0.014	< 0.001	< 0.001	< 0.001

A549					
**Drug**	**0.01 µM**	**0.1 µM**	**0.5 µM**	**0.7 µM**	**1 µM**
Erlotinib	0.033	0.009	0.006	0.002	< 0.001
Saracatinib	0.005	0.005	0.024	0.016	0.005
Dasatinib	0.003	< 0.001	< 0.001	< 0.001	< 0.001
Bosutinib	0.268	0.183	0.122	0.017	0.019

Supplementary Table S2: *P* values for survival inhibition by Src inhibitor plus erlotinib (combination vs erlotinib) or cetuximab (combination vs cetuximab) in human NSCLC cell lines, as measured by MTT assay

Src inhibitor plus erlotinib			
**Cell line**	**D1**	**D2**	**D3**
HCC827	0.666	0.045	0.002
H1975	0.176	0.070	0.140
H1299	0.330	1.000	0.448
PC-9	< 0.001	0.012	< 0.001

Src inhibitor plus cetuximab			
**Cell line**	**D1**	**D2**	**D3**
HCC827	0.003	0.006	0.004
H1975	0.276	0.006	0.002
H1299	0.739	0.011	0.013
PC-9	< 0.001	0.008	0.015

D1, D2 and D3 refer to the different doses of drugs in combinations tested in MTT assays for each cell lines.

Supplementary Table S3: Combination Index (CI) for experimental values of survival inhibition by Src inhibitor plus erlotinib or cetuximab in Ras-dependent human NSCLC cell lines, as measured by Chou and Talalay method

Src inhibitor plus erlotinib			
**Cell line**	**D1**	**D2**	**D3**
HCC827	2.441	1.537	0.837
H1975	5.769	1.255	2.549
H1299	2.007	1.855	1.749
PC-9	0.020	0.075	0.065

Src inhibitor plus cetuximab			
**Cell line**	**D1**	**D2**	**D3**
HCC827	0.121	0.022	0.028
H1975	0.295	0.094	0.043
H1299	0.662	1.102	1.091
PC-9	0.459	0.386	0.543

D1, D2 and D3 refer to the different doses of drugs in combinations tested in MTT assays for each cell lines.

Supplementary Table S4: *P* values for survival inhibition by Src inhibitor plus selumetinib (combination vs selumetinib) in Ras-dependent human NSCLC cell lines, as measured by MTT assay

Src inhibitor plus selumetinib			
**Cell line**	**D1**	**D2**	**D3**
H1299	< 0.001	< 0.001	< 0.001
Calu3-ER	< 0.001	0.006	0.002

D1, D2 and D3 refer to the different doses of drugs in combinations tested in MTT assays for each cell lines.

Supplementary Table S5: Combination Index (CI) for experimental values of survival inhibition by Src inhibitor plus selumetinib in Ras-dependent human NSCLC cell lines, as measured by Chou and Talalay method

Src inhibitor plus selumetinib			
**Cell line**	**D1**	**D2**	**D3**
H1299	0.151	0.084	0.147
Calu3-ER	0.201	0.011	0.003

D1, D2 and D3 refer to the different doses of drugs in combinations tested in MTT assays for each cell lines.
